# BNZ-gamma peptide, a potential therapeutic agent in HTLV-1 associated myelopathy

**DOI:** 10.1186/1742-4690-11-S1-P14

**Published:** 2014-01-07

**Authors:** Raya Massoud, Yoshimi Enose-Akahata, Yutaka Tagaya, Nazli Azimi, Steven Jacobson

**Affiliations:** 1 Viral Immunology Section, Neuroimmunology Branch, National Institutes of Neurological Disorders and Stroke, Bethesda, MD, USA; 2 Institute of Human Virology, University of Maryland School of Medicine, Baltimore, MD, USA; 3 BIONIZ, CA, USA

## 

The precise mechanism of HAM/TSP pathogenesis remains unclear however many findings suggest that HTLV-1 can activate the infected lymphocytes in the peripheral circulation leading to enhanced migration into the CNS. Once in this compartment, there is preferential expansion of the infected cells and a compartmentalized interaction between virus-specific CD8+ T-cells and virus- infected CD4+ lymphocytes leading to bystander damage of neural tissues. Dysregulation of T cells activation has been reported in HAM/TSP, particularly dependent on the induction of the stimulatory cytokine loops IL-2/IL-2Rα, IL-15/IL-15Rα and IL-9/IL-9Rγ by the virus. All these cytokines share a common chain receptor, the gamma chain (γC), offering a potential therapeutic target. BNZ- γ is a 19-mer peptide, that was designed based on the presence of a moderately conserved region shared by all six members of the Gamma cytokine family (IL-2, IL-4, IL-7, IL-9, IL-15 and IL-21) in their D-helix structure. *In vitro*, we showed that BNZ- γ selectively blocks binding and downstream signaling of Il-2, IL-9 and IL-15. In PBMC of HAM/TSP patients, BNZ-γ suppressed ex*–vivo* spontaneous proliferation (SP) in the majority of the tested samples. Additionally a pegylated form of the peptide (N-PEG40) could suppress SP, CD25 expression and STAT5 phosphorylation in the CD8+ T-cells. More importantly, N-PEG40 decreased proliferation of Tax-specific CD8+ cells, a known pathogenic effector cell in HAM/TSP. We are currently studying its effects on functional markers of effector /memory T-cells. These results suggest that manipulation of the inflammatory cytokine loops may be of therapeutic value in the treatment of HAM/TSP.

